# Deep-Learning-Based Model Predictive Control of an Industrial-Scale Multistate Counter-Flow Paddy Drying Process

**DOI:** 10.3390/foods13010043

**Published:** 2023-12-21

**Authors:** Ye Zhang, Zhuangdong Fang, Changyou Li, Chengjie Li

**Affiliations:** 1 College of Engineering, South China Agricultural University, Guangzhou 510642, China; zhangye@scau.edu.cn (Y.Z.); lichyx@scau.edu.cn (C.L.); 2 Shanwei Academy of Agricultural Sciences, Shanwei 516600, China; zhuangdongfang@outlook.com

**Keywords:** paddy drying, industrial-scale drying, model predictive control, computational cost, deep-learning

## Abstract

In practical industrial-scale paddy drying production, manual empirical operation is still widely used for process control. This often leads to poor uniformity in the moisture content distribution of discharged grains, affecting product quality. Model Predictive Control (MPC) is considered the most effective control method for paddy drying, but its implementation in industrial-scale drying is hindered by its high computational cost. This study aims to address this challenge by proposing a deep-learning-based model predictive control (DL-MPC) strategy for paddy drying. By establishing a mapping relation between the inlet and outlet paddy moisture content and paddy flow velocity, a DL-MPC strategy suitable for multistage counter-flow paddy drying systems is proposed. DL-MPC systems are developed using long short-term memory (LSTM) neural networks and trained using datasets from single-drying-stage and multistage drying systems. Simulation and analysis are conducted, followed by verification experiments on a 5HNH-15 multistage counter-flow paddy dryer. The results show that the DL-MPC system significantly improves computational speed while achieving satisfactory control performance. The predicted paddy flow velocity exhibits a smooth variation and matches field data obtained from multiple transition points, confirming the effectiveness of the designed DL-MPC system. The mean absolute error between the predicted and actual paddy moisture content under the DL-MPC system is 0.190% d.b., further supporting the effectiveness of the control system.

## 1. Introduction

China, as a major grain-producing country, primarily focuses on paddy cultivation [1]. In 2022, national paddy production reached 208.49 million tons [2]. The drying process is crucial for post-harvest paddy processing. However, each year in China, approximately 21 billion kilograms of grains are lost due to drying issues, resulting in spoilage and excessive fungal toxin contamination. Among these issues, the unreliable control systems and low efficiency of dryers significantly contribute to an uneven moisture distribution in dried products [3]. Addressing the global challenge of implementing an effective and reliable process control in paddy drying is crucial for minimizing post-harvest losses and ensuring food security worldwide.

Freshly harvested wet paddy often exhibits a significant variation in moisture content, causing fluctuations in grain moisture feeding in industrial-scale continuous drying equipment and making real-time prediction challenging [4,5,6]. Current control methods commonly used in practical paddy drying production still rely on manual empirical operation, which is subjective and requires specialized operators. The three main approaches for automatic control of paddy drying are feedback control, computer simulation control, and model predictive control (MPC). Feedback control, represented by proportional, integral, and derivative (PID) tuning, adjusts the drying process in real-time based on the detected grain moisture to reduce deviations [7,8], but it may not align well with the independent events of the inlet grain, drying conditions, and outlet grain in paddy drying. Computer simulation control involves modeling and solving nonlinear processes using artificial intelligence (AI) algorithms [9,10], but obtaining effective coefficients is difficult due to the randomness in field drying. Researchers have developed Boltzmann coefficient generators [11,12] that can generate real-time coefficients based on field measurements. However, this approach significantly increases control costs and presents challenges in accurately measuring data online, especially in the presence of large-scale dynamic disturbances. MPC establishes an analytical model of the drying system, utilizes disturbance information through online optimization, and calculates optimal future inputs based on the current state [13,14]. It addresses time delay effects and is suitable for controlling paddy drying that features high inertia, nonlinearity, and multiple disturbances during the dehydration process.

Researchers have developed MPC strategies specifically for grain drying, focusing on predicting and controlling the process based on heat and mass transfer mechanisms. Wu et al. [15] proposed an MPC method using temperature, humidity, and other coupled parameters represented as the equivalent moisture potential accumulation in the drying process, based on an equivalent moisture potential accumulation model. Jin et al. [16] used a data-driven approach with a back-propagation neural network to design an MPC controller that predicted the moisture content of discharged grain and adjusted the grain flow velocity to regulate the drying process. Additionally, there are hybrid MPC methods that integrate classical control approaches with data-driven strategies [17,18,19]. Despite a successful implementation in laboratory-scale drying research, applying MPC to industrial-scale drying processes is still challenging due to the high computational cost of solving the online optimization problem in real-time. This challenge arises from the nonlinear dynamic models and multi-parameter coupling involved in grain drying.

The computational cost of large-scale MPC in industrial applications is a challenge due to the need to solve nonconvex optimization problems with updated information at each prediction step [20,21]. Despite efforts to improve computational tractability [22], latency remains a limiting factor for MPC-based grain drying systems. The computational overhead can also reduce the economic benefits of investing in grain drying equipment. One potential solution to this challenge is approximating the original MPC law using function approximation techniques like deep neural networks (DNNs) [23]. By employing DNNs, the online implementation of the approximated MPC law simplifies function evaluation and significantly reduces the computational burden. Fast MPC designs using DNNs have been successful in areas like battery management [24], vehicle dynamics [25], and chemical technology [26]. However, to the best of our knowledge, there have been no previous studies that specifically explore the application of fast MPC for designing control systems in paddy deep-bed drying processes.

Multistate counter-flow paddy drying is commonly used for the centralized drying of freshly harvested wet grains [6]. It involves the grains flowing from top to bottom through multiple stages of the dryer, undergoing drying–tempering processes until they reach the desired moisture content and are discharged. This method offers benefits like energy efficiency, high productivity, and low grain breakage [27]. The height of the multistage dryer results in an extended residence time for the grains inside the drying tower. This prolonged residence time is mainly due to the need for the grains to travel through all stages of the drying process from top to bottom. It is important to carefully manage and optimize this residence time to ensure that the grains reach the desired moisture content without excessive drying or other negative impacts on their quality. When applying MPC for process control, the system needs to predict the state of the grains entering the drying system based on ambient conditions and drying parameters, including changes in inlet grain moisture content. This results in a significant computational burden for the online optimization process, which affects the real-time performance of the drying MPC system.

To address the research gap in efficient process control for multistate counter-flow paddy drying systems, this study introduces deep-learning-based MPC. Firstly, a dataset generated from classical drying MPC is used to train a deep-learning neural network that approximates the original MPC law. This approach aims to replace the optimization process of classical MPC, reducing computational costs and improving efficiency. Secondly, simulations are conducted to verify the performance of the designed controller for both single-drying stages and the multistage system. Thirdly, field experiments using a 5HNH-15 multistage counter-flow dryer validate the control performance of the proposed method. The presented paddy drying control method has practical significance for enhancing intelligent grain drying equipment in terms of transformation and upgrading.

## 2. Description of Controlled Object

**Figure 1:**
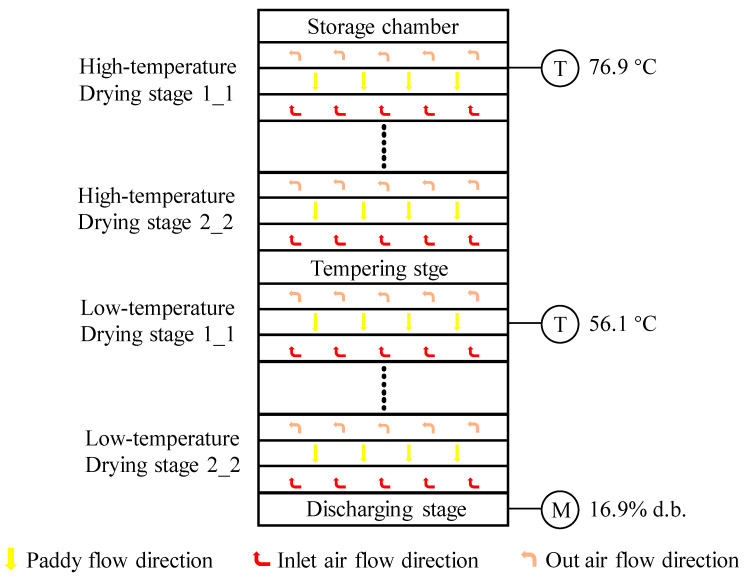
Schematic diagram of multistage counter-flow drying process.

Due to the interaction between the initial point of the counter-flow hot air and the final point of wet paddy, which corresponds to the interaction between the final point and the initial point of wet paddy, it satisfies the drying heat matching requirement where the binding energy with the moisture content decreases. This results in a better energy utilization efficiency and higher drying intensity. However, improper operation can lead to product quality issues. To alleviate internal stress in the paddy kernel and prevent breakage, a counter-flow drying process should include multiple tempering stages. Therefore, a paddy multistage counter-flow drying process often consists of multiple drying stages and tempering stages connected in series. The schematic diagram of the multistage counter-flow drying process is shown in Figure 1.

**Figure 2:**
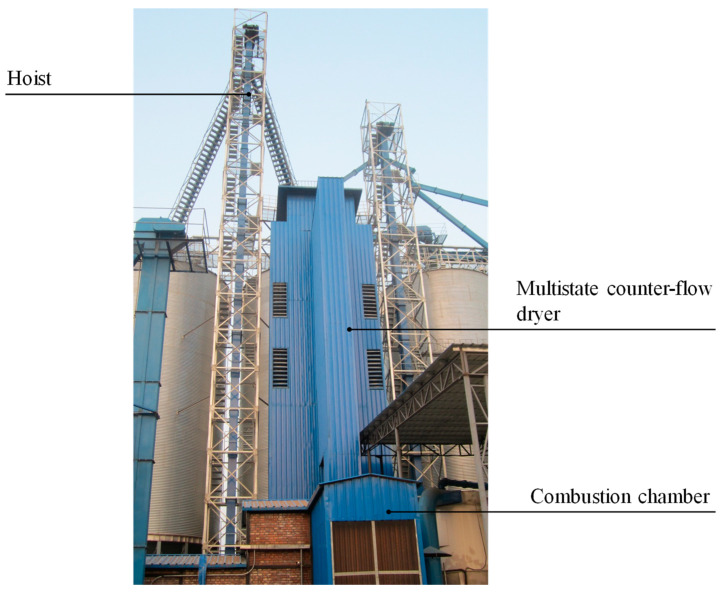
Diagram of the 5HNH-15 industrial-scale multistate counter-flow paddy dryer.

In this study, the industrial-scale multistate counter-flow paddy dryer (Model: 5HNH-15), designed by our laboratory and applied in Haina Grain Processing Center in Changsha, Hubei Province, China, was adopted as an experimental setup. The diagram of the dryer is shown in Figure 2. The total height and volume of the dryer are 14.32 m and 145.8 m^3^, respectively, and the capacity is about 90 t. The detailed drying process is as follows: Before drying, the freshly harvested wet paddy was fed into multistate counter-flow dryer by the hoist. Then, the induced fan, conveyor belt, elevator, and discharging motor were started in turn after the drying chamber was first filled with wet paddy. Paddy flowed from top to bottom of the dryer under gravity and sequentially passed through two high-temperature drying stages (with a thickness of 2 m) and two low-temperature drying stages (with a thickness of 2 m), accompanied by four tempering stages (with a thickness of 1.58 m). Finally, paddy that reached the safe moisture content was discharged from the dryer at the discharging stage.

As an aspect of the drying condition, under the action of induced fans (Model: Y4-73, Shandong Shuntong Blower Co., Ltd., Heze, China) equipped in the high- and low-temperature drying stages, low-temperature ambient air rose to the pre-set temperature after being heated by the heat exchanger, and was then fed into two drying stages, respectively. As the paddy flows downwards in the dryer, the drying air comes into contact with it and removes excess water. This moisture-laden air is then expelled from the drying system, while the partially dried paddy continues its journey towards reaching the desired moisture content.

Wet paddy undergoes a comprehensive drying process inside the dryer, which allows it to reach a safe moisture content. Throughout this period, the operator adjusts the frequency of the discharging motor in response to real-time ambient conditions, initial paddy states, drying air parameters, and operational knowledge. This adjustment modifies the paddy flow velocity, thereby changing the real-time moisture content of the discharged paddy. A detailed description of sensors and instruments that measured air temperature and humidity, paddy moisture content and temperature, and ambient state can be found in Table 1.

## 3. Construction of Controller

### 3.1. Framework of Model Predictive Control

The MPC implementation process can be briefly summarized in the following three steps: Firstly, the prediction model and current system state y_act,i_ are utilized to predict the output y_act,i+1_, y_act,i+2_, …, y_act,tp−1_, y_act,tp_ of the future time domain t_p_. Secondly, under system constraints, a specific optimization method is adopted to determine an input sequence u_i+1_, u_i+2_, …, u_tc−2_, u_tc−1_ that satisfies the objective function within a control time domain, aiming to achieve an actual output that closely approximates the objective output y_obj_ in future time domain t_p_. Thirdly, the first element of the obtained input sequence, u_i+1_, is applied to the control system to obtain the system state at time i + 1. Then, the current time is set as i, and the above steps are repeated. As the control system operates, the prediction time domain continuously rolls forward to achieve control over the entire operation process. Specifically, the above optimization problem can be illustrated in Equation (1) [28]:

(1) $$J=\sum_{i=1}^{t_{p}}Q{\left(y_{obj}-y_{act,i}\right)}^{2}+\sum_{i=1}^{t_{p}}R\Delta u_{i}{}^{2}$$

where Q and R are the weighting matrices.

**Figure 3:**
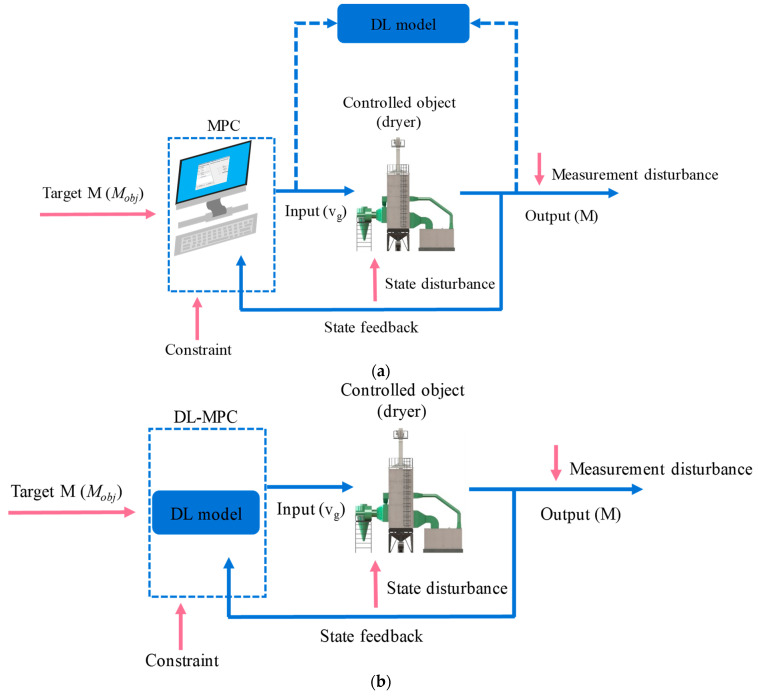
Schematic diagram of multistage counter-flow drying process: (**a**) train process and (**b**) application process.

The above description for MPC illustrates that the essence of MPC is to obtain the future input based on a prediction model and measured system state data, wherein the actuator is the selected optimizer. To address the computational burden of classical MPC systems and enable their practical application in the field, the mapping relation between measurable state variables and control inputs can be established. This approach replaces the online optimization process in the classical MPC system, making it a more intuitive means of implementing MPC in field applications. Based on establishing a complete and accurate mechanistic simulation model for the drying system [6,29], a deep learning method can establish a mapping relation from state to action using a large amount of simulation data. This allows MPC to be implemented without the need for online optimization. The principle and primary implementation method are shown in Figure 3. The output in typical MPC (M in Figure 3a) is selected as the input of the deep-learning model, and the input in typical MPC (v_g_ in Figure 3a) is selected as the output. Finally, a deep-learning-based optimizer, as shown in Figure 3b, can be obtained to replace the MPC in Figure 3a.

### 3.2. Deep-Learning-Based MPC

During the paddy drying process, the moisture content of the paddy discharged from the dryer is affected by fluctuations in the ambient state, drying air state, and machine operation. These factors influence the paddy as it flows through the complete drying tower. Therefore, the output, which is the moisture content of the discharged paddy, at a given time is not solely determined by the current input factors such as the ambient or drying air state. It also depends on past inputs that have occurred over a certain period. As a result, modeling a paddy drying system using data-driven approaches can be considered as modeling time series data, considering the temporal dependencies and patterns in the system. The Recurrent Neural Network (RNN), incorporating temporal dependence into the traditional feedforward neural network, introduces the previous output as an input to the next hidden layer. This unique structure gives the RNN short-term memory capabilities and preserves the relations among data, making it suitable for analyzing time series data [30]. The long short-term memory (LSTM) model, a variant of the RNN, addresses issues such as gradient vanishing/exploding and limited long-term memory capacity [31].

**Figure 4:**
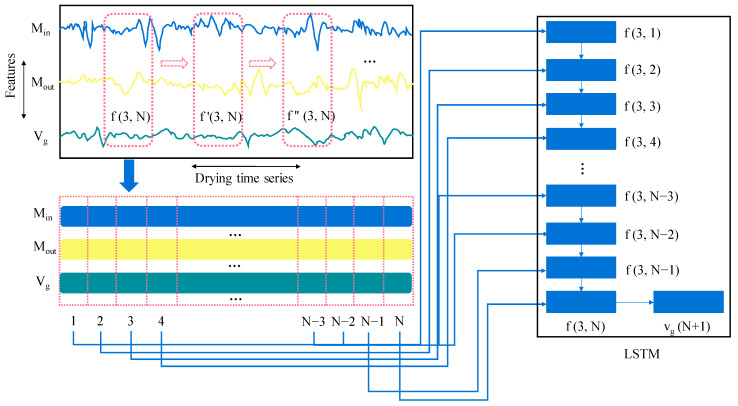
Diagram and application process of paddy drying LSTM predictive controller.

The application process of the LSTM-based predictive controller is illustrated in Figure 4. It involves taking a time-sliding window of system state variables and input variables, f(3, N), which are collected in a preserved time sequence order and fed into an LSTM network as inputs to predict the next moment’s system input, v_g_(N + 1), where N represents the length of the time series. As the drying progresses, the sliding window moves forward continuously, capturing f′(3, N), f″(3, N), and so on. The LSTM utilizes the input data to calculate the output for the next moment and transfers it to the drying system, completing the predictive control process. In this study, the selected system state variables are the inlet moisture content (M_in_) and outlet moisture content (M_out_), while the input variable is the paddy flow velocity (v_g_). In this framework, adjusting the paddy flow velocity takes into account not only the current system state but also the historical states and inputs within a specified time period. This approach goes beyond a simple one-to-one correspondence between input and output at a single moment. By considering a broader range of information, it allows for a more comprehensive, extensive, and accurate understanding of the system dynamics.

### 3.3. Analytical Model for Data Generation and Collection

This study combines numerical simulation and experimental research to obtain a comprehensive dataset for constructing DL-MPC. Specifically, an analytical model based on heat and mass transfer mechanisms is adopted to simulate various drying conditions in paddy drying processes. It should be noted that the predictive reliability of the mathematical model needs to be validated. In our previous research [6,32], we developed a mathematical model for paddy drying based on the spatial description method, which was successfully applied to analyze the process of the paddy multistage counter-flow dryer, the focus of this study. For the drying stage where water removal occurs, the mathematical model can be represented by the following set of partial differential equations:

(2) $$\rho_{g}\frac{\partial M_{d}}{\partial t}-v_{g}\rho_{g}\frac{\partial M_{d}}{\partial Z}=-\mu \gamma \alpha (d_{s}-d)\frac{M_{d}-M_{e}}{M_{0}-M_{e}},$$

(3) $$c_{g}\rho_{g,w}\frac{\partial T_{g}}{\partial t}+v_{a}c_{g}\rho_{g,w}\frac{\partial T_{g}}{\partial Z}=-h\tau \alpha (T_{a}-T_{g})-\lambda_{g}\mu \gamma \alpha (d_{s}-d)\frac{M_{d}-M_{e}}{M_{0}-M_{e}},$$

(4) $$v_{a}\rho_{a}\frac{\partial d}{\partial Z}=\mu \gamma \alpha (d_{s}-d)\frac{M_{d}-M_{e}}{M_{0}-M_{e}},$$

(5) $$c_{a}\rho_{a}v_{a}\frac{\partial T_{a}}{\partial Z}=-h\tau \alpha (T_{a}-T_{g})$$

where *ρ_g_* is the bulk density of absolute dry paddy, *M_d_* is the paddy moisture content, on a dry basis, *t* is the drying time, *v_g_* is the paddy flow velocity, *Z* is the bed height, *μ* is the interphase heat transfer coefficient, *γα* is the effective evaporation area of unit volume, *d_s_* is the saturated humidity, *M* is the moisture content on a dry basis, *c_g_* is the specific heat of paddy, *v_a_* is the drying air flow velocity, *T* is temperature, *λ_g_* is the latent heat of vaporization of paddy, *ρ_a_* is the air density, *h* is the heat transfer coefficient, *τα* is the effective heat transfer area per unit volume, and *ρ_g,w_* is the bulk density of wet paddy.

The tempering stage that facilitates the even distribution of temperature and water in a kernel can be represented by the following mathematical model:

(6) $$\frac{\partial M_{d}}{\partial t}=v_{g}\frac{\partial M_{d}}{\partial Z},$$

(7) $$\frac{\partial T_{g}}{\partial t}=v_{g}\frac{\partial T_{g}}{\partial Z}-S(T_{g}-T_{ab})$$

where *S* is the cooling coefficient.

The numerical solution method is employed to solve the aforementioned mathematical model. The initial and boundary conditions can be represented as follows. 

Initial conditions:

(8) $$\left\{\begin{array}{l} M_{i,0}=M_{0}\\ T_{g,}{}_{i,0}=T_{g,0} \end{array}\right.$$

Boundary conditions:

(9) $$\left\{\begin{array}{l} d_{0,t}=d_{0}\\ T_{a,}{}_{0,t}=T_{a,0}\;, \end{array}\right.$$

where *i* refers to the *i*th layer of the mesh divided in the z-direction.

Some property values and equations used in the solving process are listed in Table 2. We have conducted multiple model validation experiments in our previous research [32], and the results showed that the root-mean-square error (RMSE) for predicting paddy drying moisture content and temperature is 0.98% d.b. and 0.49 °C, respectively. The mean relative deviation (MRD) is 5.5% for paddy moisture content and 1.42% for temperature. These reasonable reliability indicators demonstrate the effectiveness of the mathematical model in analyzing actual paddy processes.

### 3.4. Statistical Analysis

In this study, the mean absolute error (MAE), defined in Equation (10), was adopted to evaluate the predictive accuracy of DL-MPC to paddy flow velocity when applied in the multistate counter-flow dryer:

(10) $$MAE=\frac{1}{n}\sum_{i=1}^{n}\left|y_{pre}-y_{obsj}\right|$$

where y_pre_ and y_obsj_ are the predicted and experimental values, respectively, and n is the number of measurements for each experiment.

## 4. Simulation

### 4.1. Simulation of Single-Drying Stage

The drying stage is the key phase in a multistage counter-flow dryer, where the primary focus is reducing moisture content during the paddy drying process. The tempering stage primarily regulates the paddy kernel temperature and does not involve moisture removal; it functions as an auxiliary stage. In this section, we focus on studying a single drying stage. We simulate the paddy drying process in a single-core counter-flow drying stage. The objective is to observe the response capability of the DL-MPC system when there are changes in the inlet paddy moisture content. States of the ambient conditions, drying system, wet paddy, and drying air are listed in Table 3. It should be noted that the variations in process parameters occur after the system reaches a steady state, i.e., after the wet paddy that has not undergone the complete drying process in the first tower has been discharged from the drying system.

**Figure 5:**
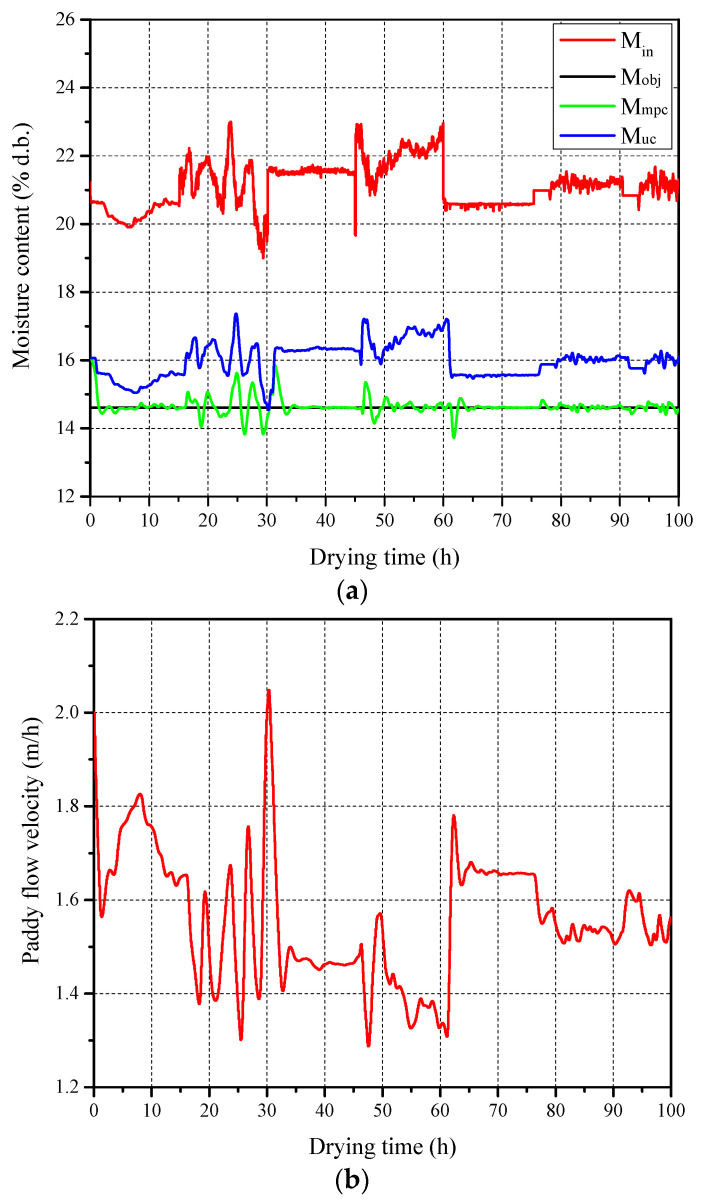
Dataset for building DL-MPC: (**a**) Paddy moisture content and (**b**) flow velocity.

The variation in inlet paddy moisture content is the crucial factor affecting the drying efficiency of multistage counter-flow dryers. To provide sufficient information for training a neural network controller, this study defines an inlet moisture content signal sequence based on actual drying experimental data and past field operation experience, as shown in Figure 5. The defined inlet moisture content signal sequence (M_in_) has a duration of 100 h with a sampling period of 0.01 h, generating 10,000 data points within the range of 19–23% d.b. It thoroughly analyzes practical process disturbances, including step changes, sinusoidal variations, and noise, to deliver valuable identification information. Under the designed classic MPC system (Figure 3a), the discharging paddy moisture content is controlled to approach the target moisture content by continuously adjusting the paddy flow velocity (Figure 5b). As shown in Figure 5a, compared with the uncontrolled moisture content curve (M_uc_), the MPC-controlled moisture content curve (M_mpc_) is primarily distributed around the target moisture content curve (M_obj_), with an MAE of 0.132% d.b.

After standardizing the dataset obtained from the classical MPC system, the data consisting of 10,000 samples were divided into training and test sets in an 8:2 ratio. The training set was employed to fit a neural network, while the test set was used for evaluating the model’s generalization capability. The input for the LSTM model, developed using the Keras library in Python’s TensorFlow 2.0 deep-learning framework, consisted of the inlet paddy moisture content, paddy flow velocity, and outlet paddy moisture content in the preceding 10 time steps (N = 10). The model output was the predicted paddy flow velocity for the subsequent time step. Specifically, the LSTM architecture for forecasting paddy flow velocity in a single counter-flow drying stage comprised two layers, with each layer comprising 50 units. During the model training process, the Adam optimization algorithm was utilized with a batch size of 20, a dropout rate of 0.2, and a time step length of 30.

**Figure 6:**
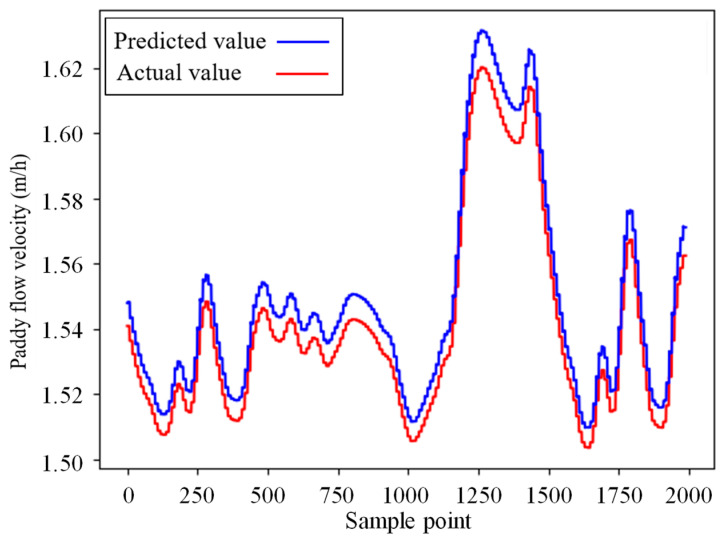
Prediction results of model on the test set of single-drying stage simulation.

Upon reaching 11 epochs, the neural network prediction model exhibited a stable mean square error (MSE) and determination coefficient (R^2^) values. After completing 30 epochs, training concluded with an MSE below 0.0005 and an R^2^ exceeding 0.98. Utilizing this trained model, predictions were made on the test set and compared to actual values of paddy flow velocity in Figure 6. The curves of predicted and actual values closely aligned, indicating a strong resemblance. The mean absolute error (MAE) was 0.006 m/h, while R^2^ was 0.937, showcasing the LSTM neural network’s high accuracy in forecasting and its generalizability.

**Figure 7:**
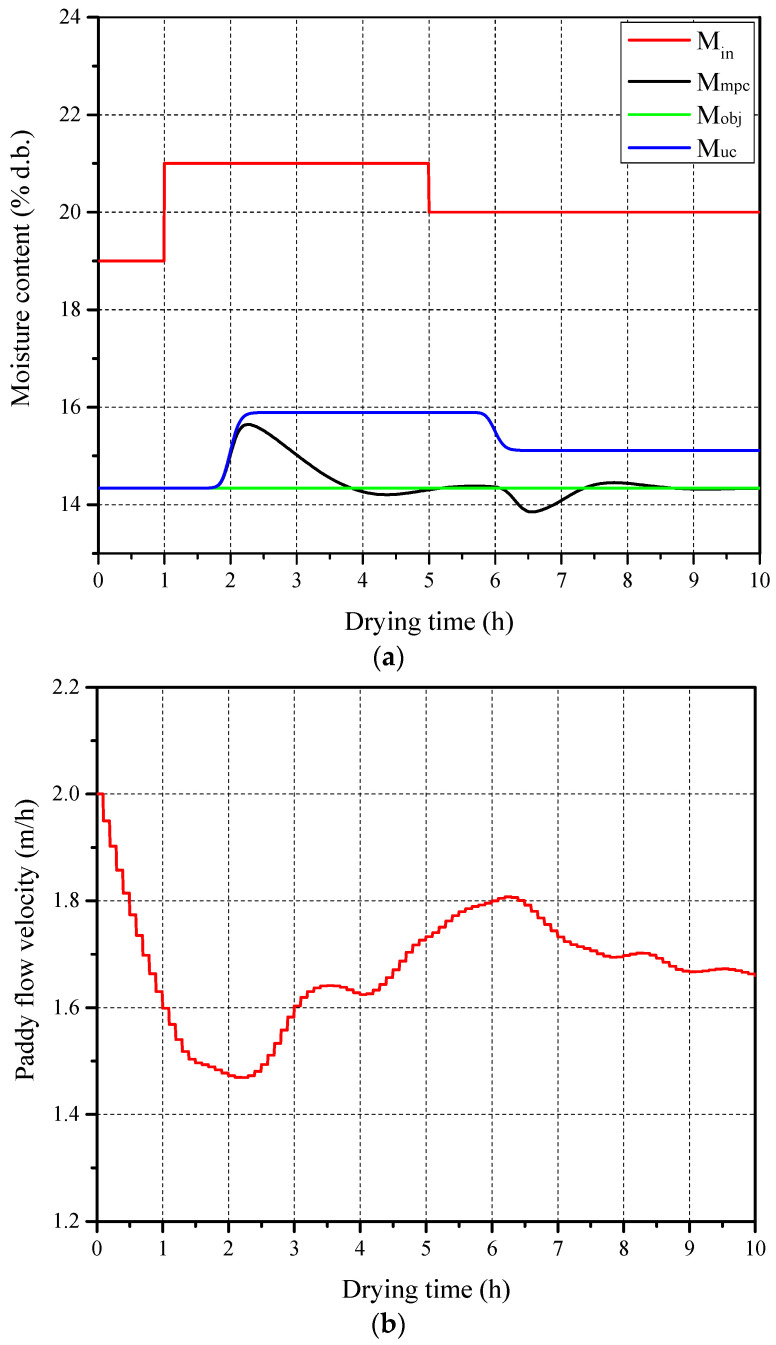
Test result of DL-MPC of single drying stage: (**a**) Paddy moisture content and (**b**) flow velocity.

The obtained LSTM neural network model was imported into the MATLAB 2017b environment to replace the optimization process of the classical MPC and to construct a DL-MPC simulation system. This system was used to observe the response capability of the DL-MPC system to variations in paddy initial moisture content. The basic parameters for the ambient state, drying system, paddy, and initial state of drying air were taken from Table 2. The simulation results are presented in Figure 7. It can be observed that the DL-MPC system made corresponding adjustments whenever there was a sudden change in the inlet paddy moisture content. The simulation time for the DL-MPC system, running on the simulation software and hardware platform (Intel(R) Core CPU i5-11320 running at 3.2 GHz) under the drying conditions corresponding to a drying time of 10 h, was 7.245 s. In contrast, the simulation time for the classical MPC system under the same drying conditions was 144.889 s. This demonstrates that compared with the classical MPC, the DL-MPC system significantly improves computational speed while ensuring the completion of control tasks, making it more suitable for online processes.

### 4.2. Simulation of Multiple-Drying Stage

Based on the construction and simulation of the single-stage DL-MPC system, a similar approach can be used to build a multistage counter-flow drying DL-MPC system. By extending the methodology to multiple stages, we can develop a comprehensive control system that optimizes the drying process across all stages in the counter-flow dryer. This allows for improved control and efficiency throughout the entire drying operation. In this section, the response capability of the DL-MPC system to variations in the inlet paddy moisture content is observed during the paddy drying process in the multistage counter-flow drying system shown in Figure 1. Table 4 presents the fundamental parameters of the ambient state, drying system, paddy, and initial state of the drying air. Similarly, changes in process parameters occur after the system reaches a steady state.

**Figure 8:**
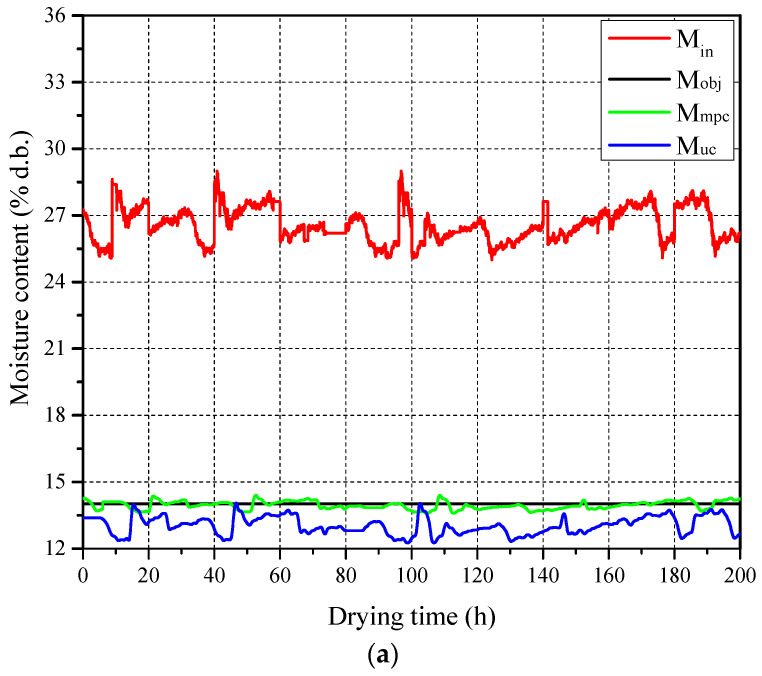

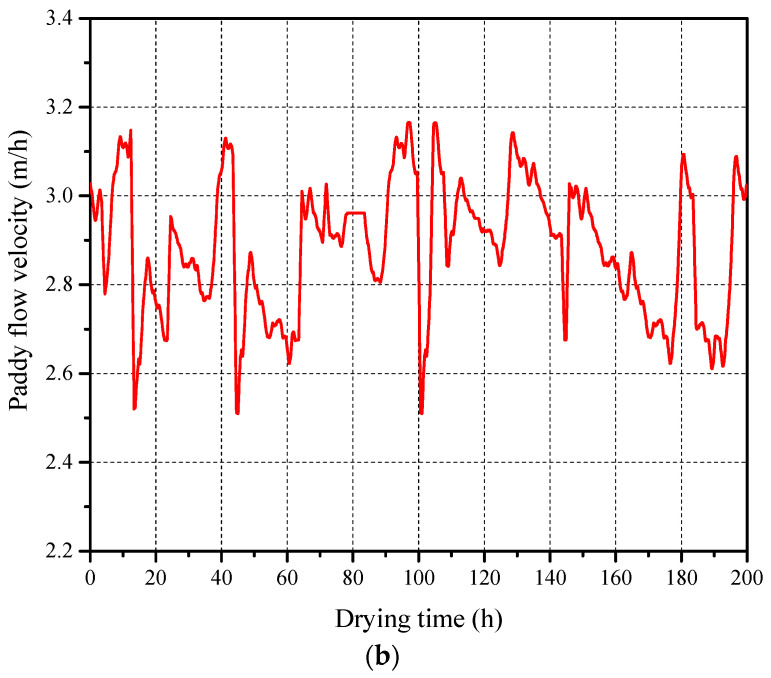
Dataset for constructing DL-MPC of multistage counter-flow drying system: (**a**) Paddy moisture content and (**b**) flow velocity.

In the multistage counter-flow drying system, the paddy drying process is more complex and influenced by numerous factors. Thus, compared with a single drying stage, more data are required to construct a training set with abundant features. As shown in Figure 8a, a fluctuating signal sequence of inlet paddy moisture content was defined for 200 h with a sampling interval of 0.01 h, resulting in 20,000 data points within the range of 25–29% d.b. amplitude. From Figure 8b, it can be observed that the classical MPC system, when faced with variations in the inlet paddy moisture content, can continuously adjust the paddy flow velocity to maintain the outlet paddy moisture content around the target value, with an MAE of 0.161% d.b.

**Figure 9:**
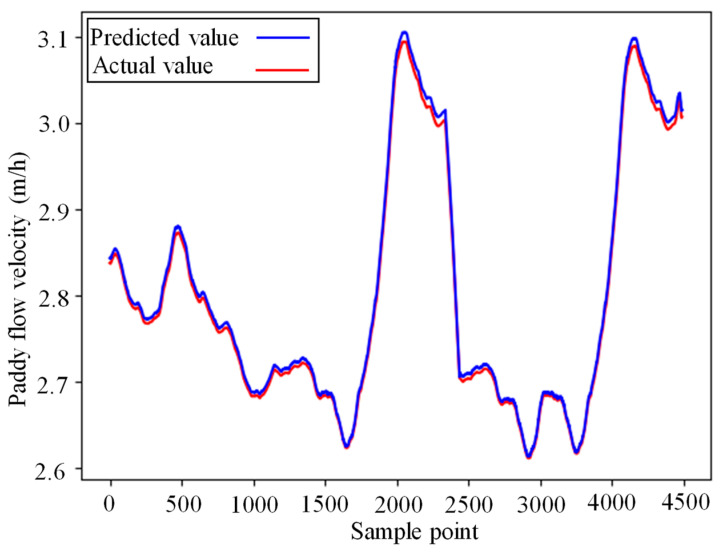
Prediction results of model on the test set of multiple-drying stage simulation.

After standardizing the database obtained from the classical MPC system, a similar approach as the single drying stage was used to train an LSTM network. In the model training process, N was set to 70. The LSTM network consisted of two layers, each with 50 units. The Adam optimization algorithm was utilized for model training, with a batch size of 20, a dropout rate of 0.2, and a time step length of 30. After reaching 13 epochs, the neural network prediction model’s MSE and R^2^ tended to stabilize. By the time the training reached 30 epochs, the training was completed, with an MSE below 0.0005 and an R^2^ exceeding 0.98. The trained neural network model was then used to predict the data on the test set, and the predicted paddy flow velocity was compared with the actual values, as shown in Figure 9. It can be seen that the predicted and actual curves of the paddy flow velocity closely overlap. The MAE was found to be 0.0058 m/h, and the R^2^ was 0.9980, indicating that the established LSTM neural network demonstrated high prediction accuracy and good generalization capability.

**Figure 10:**
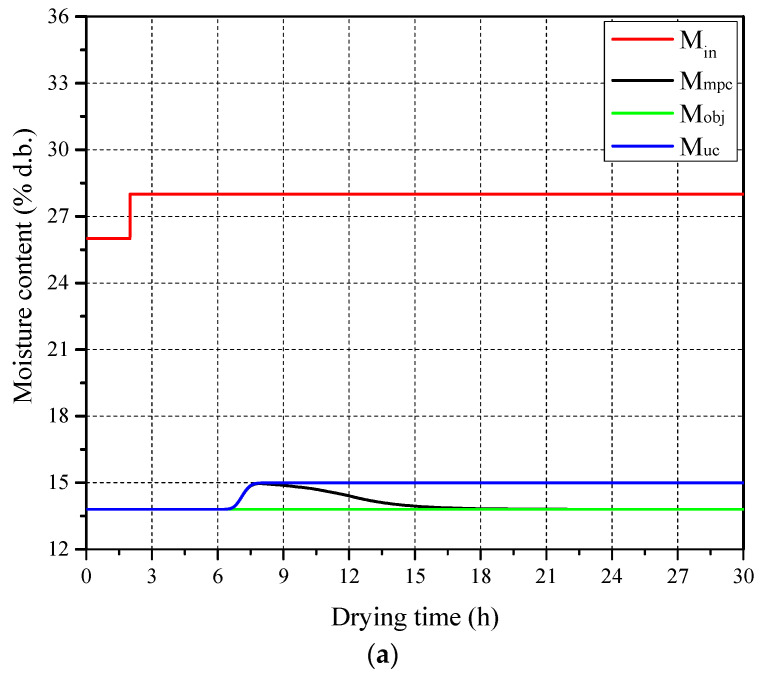

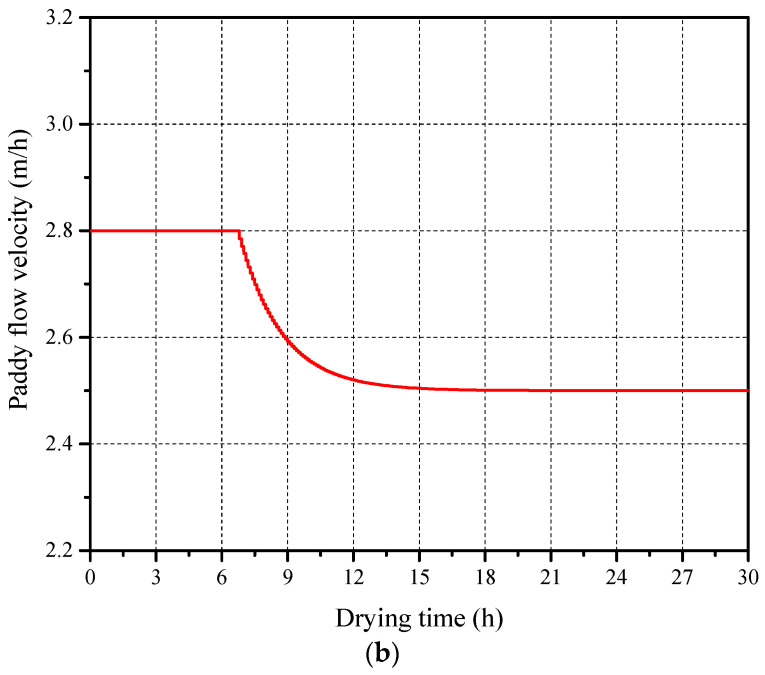
Test result of DL-MPC of multistate drying: (**a**) paddy moisture content and (**b**) flow velocity.

The LSTM neural network model was imported into the MATLAB environment to replace the optimization process of classical MPC, creating a DL-MPC simulation system for paddy multistage counter-flow drying. The DL-MPC system’s response to variations in the inlet paddy moisture content was observed using the basic parameters provided in Table 3 for the ambient state, drying system, paddy, and initial state of the drying air. Figure 10 presents the simulation results which demonstrate that the DL-MPC system made appropriate adjustments whenever there was a sudden change in the inlet paddy moisture content. The simulation period for the DL-MPC system was 22.499 s for a drying time of 30 h, while the classical MPC took 15,120 s under the same drying conditions. This significant improvement in computational speed enables the DL-MPC system to effectively accomplish control tasks in online processes, making it more suitable for such applications. 

Furthermore, from Figure 10a, it can be observed that, due to the single adjustment control method used in this study and the larger height of the multistage counter-flow drying system, the system’s adjustment time is longer. When the drying air parameters and paddy flow rate are adjusted simultaneously, the system’s adjustment time might be shorter. Therefore, in future research, the utilization of multiple adjustment control variables will be explored. This includes simultaneously adjusting variables such as drying air temperature, drying air flow velocity, and paddy flow velocity, with the aim of constructing a multi-variable DL-MPC controller.

## 5. Experiment

**Figure 11:**
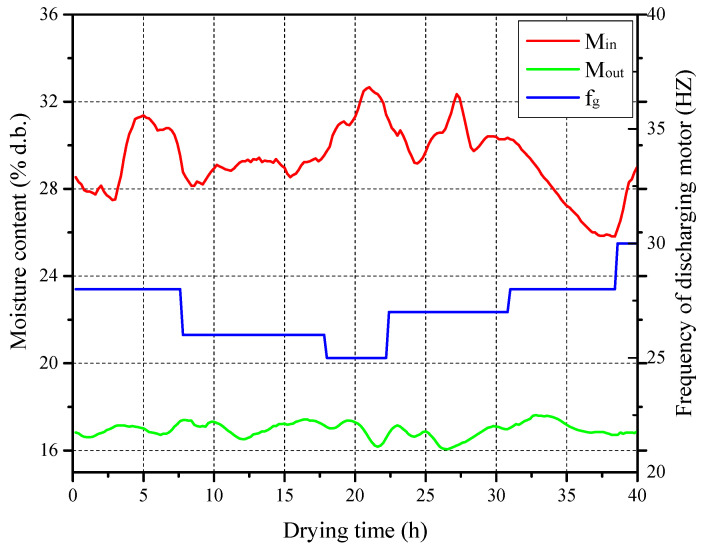
Real-time moisture content of inlet and outlet grain and frequency of grain discharging motor during drying process.

In this section, the performance of the designed DL-MPC system was analyzed through experiments using the 5HNH-15 laboratory-designed multistage counter-flow dryer shown in Figure 2. The structural parameters of the 5HNH-15 dryer, as well as the data acquisition system and sensor parameters, are provided in Section 2. The experimental paddy sample used was Guangliangyouxiang 66. During the data collection period, the average ambient temperature and relative humidity were 9.6 °C and 66.6%, respectively. The average inlet air temperature in high- and low-temperature stages were 76.9 °C and 56.1 °C, respectively. Over the 40 h test period, real-time measurements of the inlet paddy moisture content, outlet paddy moisture content (M_out_), and frequency of the grain discharging motor (f_g_) are shown in Figure 11.

**Figure 12:**
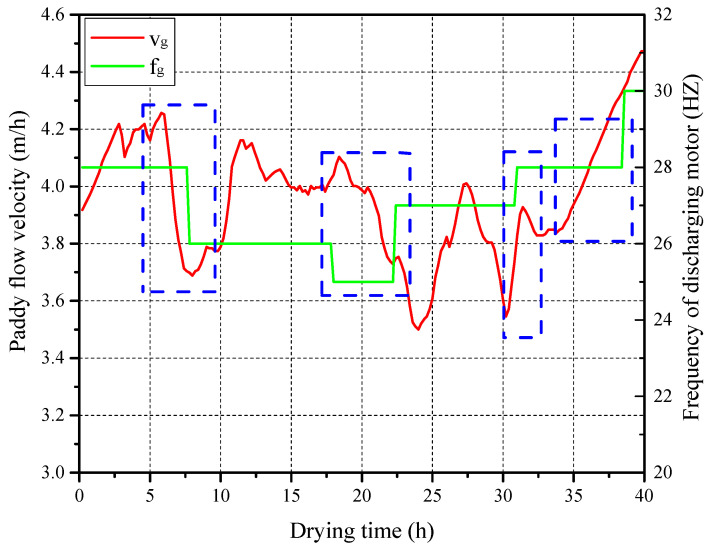
Predicted grain flow velocity and frequency of grain discharging motor.

During the construction of the training dataset for the DL-MPC system, to reduce data quantity and facilitate the feasibility testing of the proposed method, it was assumed that the ambient state and ventilation parameters remained constant (taken as the average measured values). Based on field experimental data, the inlet paddy moisture content dataset was generated. The DL-MPC system was then constructed using the controller design method described in Section 3, followed by simulation. The predicted paddy flow velocity was compared with the actual frequency of the discharging motor. The results are shown in Figure 12. It can be observed that compared with the actual frequency of the discharging motor obtained from the field, the predicted paddy flow velocity exhibits a smooth variation and shows the same changing trend as the actual frequency of the discharging motor at multiple transition points (highlighted in blue boxes in Figure 12), indicating the effectiveness of the designed DL-MPC system. However, since the DL-MPC system ignores the variations in the ambient state and fluctuations in the inlet drying air conditions, the paddy flow velocity under DL-MPC strictly follows the changes in inlet paddy moisture content, resulting in some differences between the predicted data trends and the field data.

**Figure 13:**
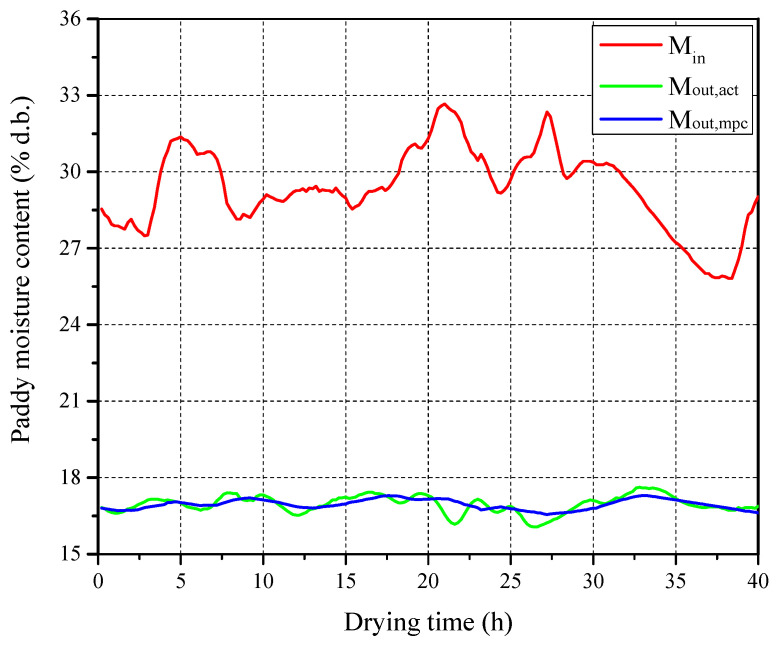
Comparison between predicted and actual moisture content.

The predicted outlet paddy moisture content (M_out,mpc_) obtained from the DL-MPC system is compared with the actual values (M_out,act_), and the results are shown in Figure 13. It can be observed that compared with the actual outlet paddy moisture content, the predicted values are distributed more consistently around the target moisture content, with smaller variations as the inlet paddy moisture content changes. This is because the DL-MPC system has a higher adjustment frequency and more precise control than manual adjustments. The MAE between the predicted and actual values of the outlet paddy moisture content is 0.19% d.b.

## 6. Discussions

When the goal is to maintain a safe moisture content in paddy drying systems, the control problem can be addressed by rejecting disturbances and ensuring zero steady-state error in the system output. However, the significant lag response characteristics of drying systems limit the effectiveness of relying solely on output feedback control [34,35]. By considering the overall system state and incorporating modern control techniques like state observation, optimal state estimation, and system model parameter identification, it is possible to achieve disturbance rejection control in drying systems using model predictive control principles. Our DL-MPC control system stands out for its ability to provide a rapid response by replacing the optimization process in MPC with a deep-learning-based data mapping function. Through simulation experiments, we demonstrate that the calculation of input parameters for drying processes lasting over 10 h can be completed within 10 s. In contrast to previous approaches primarily using data-driven models instead of mechanistic models for controller optimization, which still rely on iterative processes [36,37], our method offers a different perspective. Jin et al. [38] proposed an approach integrating LSTM neural networks to approximate the actual drying process and constructed an LSTM-MPC controller for dryer control, with a response time of 1150 s. Dai et al. also attempted to design grain drying controllers using machine learning methods, such as genetic optimization algorithms [39] and support vector machines [40]. While these small-sample modeling approaches improve computational speed to some extent, their control precision heavily relies on the accuracy of the data-driven model. In contrast, our DL-MPC system achieves an acceptable accuracy in controlling the target moisture content, with a MAE of 0.19% d.b. Another study conducted by Li et al. [14] proposed an NARX-based PSO-MPC controller for counter-current flow grain dryers, achieving an acceptable control accuracy of 0.52%, but this approach also faces challenges related to high data dependency. Similar results have been reported in MPC drying experiments with agricultural products like sawdust [41], corn [42], and red maple [43].

**Figure 14:**
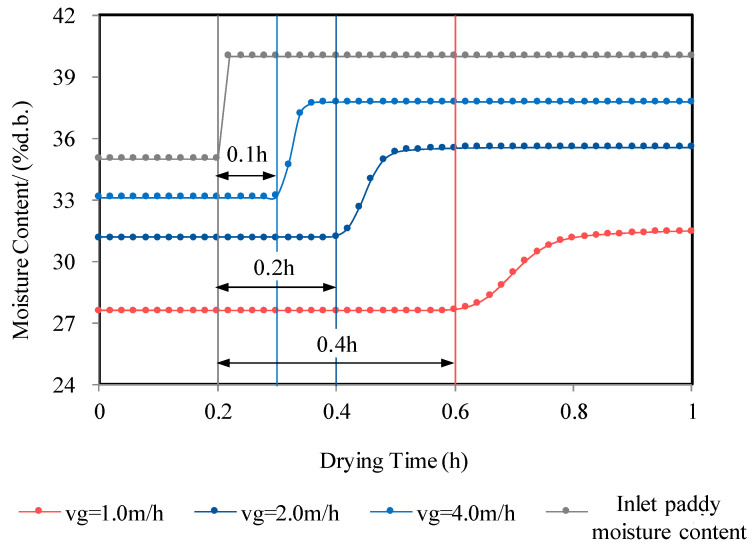
Transient response of counter-flow drying system with inlet paddy moisture content step change.

On the other hand, the unique material properties of paddy make its deep-bed drying process different from that of vegetables, fruits, and other agricultural products. It should be noted that the significant time delay characteristic of drying systems poses an important challenge in designing a paddy drying controller [6,44]. Although the controller designed for the multistage counter-flow dryer can achieve accurate process regulation, the adjustment time is excessively long, possibly exceeding 6 h. Figure 14 illustrates the transient response characteristics of paddy drying with step changes in grain moisture content at different paddy flow velocities, focusing on a single drying stage mentioned in Section 4.1. The figure shows that when the drying time is 0.2 h, the paddy moisture content jumps from 35% d.b. to 40% d.b., and the system transitions from one steady-state point to another, with a delay before the output begins to respond. For a paddy flow velocity of 1 m/h, the delay time is 0.4 h, the time for paddy to flow within the layer is 0.5 h, and the transition time is 0.6 h. Increasing the paddy flow velocity to 2 m/h reduces the delay time to 0.2 h, the flow time within the layer to 0.25 h, and the transition time to 0.3 h. Similarly, a paddy flow velocity of 4 m/h further decreases the delay time to 0.1 h, the flow time within the layer to 0.125 h, and the transition time to 0.15 h. In the step change process of grain moisture input, the transition time is greater than the flow time, which in turn is greater than the delay time. Additionally, higher paddy flow velocities require shorter delay and transition times. This analysis reveals that the flow-layer drying system exhibits pure lag characteristics, where information propagation is influenced by the time it takes for paddy to pass through the drying layer. Slower paddy flow velocities result in slower information propagation and longer system output delays. Relying solely on output feedback control for a system with pure lag characteristics leads to a significant overshoot, longer adjustment time, and poor control performance [45]. These findings align with previous studies that concluded that output feedback PID controllers [46,47] are not suitable for deep-bed flow-layer drying systems.

## 7. Conclusions

This study addresses the high computational cost challenge of applying classical MPC to industrial-scale paddy drying. A deep-learning-based model predictive control strategy suitable for paddy multistage counter-flow drying systems is proposed and designed through numerical simulation and optimization design. The controller’s performance is validated through online and field experiments, leading to the following conclusions:

(1) For a single-drying stage, the DL-MPC system achieves a simulation time of 7.245 s, significantly improving computational speed compared to classical MPC (144.889 s). This makes DL-MPC more suitable for online processes while ensuring effective control.
(2) In multistage combined continuous drying, DL-MPC reduces the control time from 15,120 s to 22.499 s. However, due to the tall height of the control object, the adjustment time is longer compared to a single-drying stage, indicating the pure lag characteristics of the paddy deep-bed drying system.
(3) Field validation experiments demonstrate that the predicted paddy flow velocity follows the discharge motor frequency trend and exhibits a smoother variation. This indicates that the designed DL-MPC controller offers a higher adjustment frequency and more precise control compared to manual adjustments. The MAE between the predicted and actual outlet paddy moisture content is 0.19% d.b., confirming the effectiveness of the DL-MPC controller.

Considering the significant time delay characteristics in multistage counter-flow drying systems, future research will explore the utilization of multiple adjustment control variables, such as simultaneously adjusting the drying air temperature, drying air temperature flow velocity, and paddy flow velocity, to construct a multi-variable DL-MPC controller.

## Figures and Tables

**Table 1:** Detailed description of the sensors and instruments.

Devices	Model	Precision	Manufacturer
Air convection oven	DHG070B	-	Shanghai Anting Scientific Instrument Factory, Shanghai, China
Infrared thermometer	62 MAX+	1 °C	Fluke Testing Instruments (Shanghai) Co., Ltd., Shanghai, China
Temperature and humidity sensor	AM2305	0.3 °C and 2%	Guangzhou Aosong Electronics Co., Ltd., Guangzhou, China
Anemometer	AS8336	±0.01 m/s	Guangzhou Ximarui Electronics Co., Ltd., Guangzhou, China
Electronic scale	DY-718	±1 g	Jinhua Furuisi Electronics Co., Ltd., Jinhua, China

**Table 2:** Thermodynamic equations and property values used in calculation process.

Meters	Equations/Values	Units
*P_s_*	$P_{s}=133.322\exp(18.751-\frac{4075.16}{236.516+T_{ab}})$	Pa
*d*	$d=0.622\cdot \frac{RH\cdot P_{s}}{P_{b}-RH\cdot P_{s}}$	kg/kg
*M_e_*	$M_{e}={\left[\frac{-\ln(1-RH)}{1.919\times 10^{-5}(T_{a}+51.161)}\right]}^{\frac{1}{2.445}}$	g water/g dry matter
*ρ_a_*	1.293	kg/m^3^
*c_a_*	1.005	kJ/(kg·°C)
*ρ_g_*	$\rho_{g}=\frac{1}{1+M_{d}}(2.073M_{d}+508.5)$	kg/m^3^
*c_g_*	$c_{g}=1.11+0.045\frac{M_{d}}{1+M_{d}}$	kJ/(kg·°C)
*λ_g_*	$\lambda_{g}=[2500-2.386(T_{g}+273)][1+2.556\exp(-20.176M_{d})]$	kJ/kg
*μγα*	$\mu \gamma \alpha =\frac{\rho_{g}r(M_{d}-M_{e})}{\left(d_{s}-d\right)}$	kg/(h·m^3^)
*r*	$r=0.0153T_{a,in}-0.215$	1/h
*hτα*	$h\tau \alpha =V_{a}c_{a}\rho_{a}\frac{T_{a}{{}_{,}}_{in}-T_{a,}{}_{out}}{Z(\bar{T_{a}}-\bar{T_{g}})}$	kJ/(h·m^3^·°C)

Note: The atmospheric pressure P_b_ is 101,325 Pa in this study. R is drying constant. The equation for calculating r is quoted from the research by Motohashi and Hosokawa [33].

**Table 3:** Parameters of drying system.

Parameters	Values	Units
Thickness of drying stage	0.5	m
Thickness of tempering stage (air outlet)	0.5	m
Target moisture content	14.61	% d.b.
Paddy initial temperature	20	°C
Ambient temperature	20	°C
Ambient relative humidity	50	%
Drying air temperature	60	°C
Drying air flow velocity	2300	m/h
Controllable range of paddy flow velocity	1–5	m/h

**Table 4:** Parameters of multistage counter-flow drying system.

Parameters	Values	Units
Thickness of drying stage	0.5	m
Thickness of tempering stage (air outlet)	0.5	m
Thickness of tempering stage	1.58	m
Target moisture content	14.01	% d.b.
Paddy initial temperature	20	°C
Ambient temperature	20	°C
Ambient relative humidity	50	%
Drying air temperature in high-temperature drying stage	70	°C
Drying air temperature in low-temperature drying stage	50	°C
Drying air flow velocity in high-temperature drying stage	1924.5	m/h
Drying air flow velocity in low-temperature drying stage	1154.2	m/h
Controllable range of paddy flow velocity	1–5	m/h

## Data Availability

Data are contained within the article.
